# A diagnostic accuracy study on an innovative auto-edge detection technique for identifying simulated implant fractures on radiographic images

**DOI:** 10.1038/s41598-022-24266-7

**Published:** 2022-11-16

**Authors:** Negar Khosravifard, Bardia Vadiati Saberi, Amir Khosravifard, Hamidreza Zakerjafari, Reihaneh Vafaei, Mohammad Ebrahim Ghaffari

**Affiliations:** 1 Department of Oral and Maxillofacial Radiology, Dental Sciences Research Center, School of Dentistry, Guilan University of Medical Sciences, Rasht, Iran; 2 Department of Periodontics, Dental Sciences Research Center, School of Dentistry, Guilan University of Medical Sciences, Rasht, Iran; 3 Department of Mechanical Engineering, Shiraz University, Shiraz, Iran; 4 Department of Dental Prosthesis, Dental Sciences Research Center, School of Dentistry, Guilan University of Medical Sciences, Rasht, Iran; 5 Department of Biostatistics, Dental Sciences Research Center, School of Dentistry, Guilan University of Medical Sciences, Rasht, Iran

**Keywords:** Medical research, Experimental models of disease

## Abstract

Implant fracture is a rare but devastating complication of treatment in partially or fully edentulous patients which requires prompt diagnosis. Nevertheless, studies on defining the most accurate technique for the detection of implant fractures are lacking. In the present study, the Canny edge detection algorithm was applied on multiple radiographic modalities including parallel periapical (PPA), oblique periapical (OPA), and cone beam CT (CBCT) with and without metal artifact reduction (MAR) to examine its accuracy for diagnosis of simulated implant fractures. Radiographs were taken from 24 intact implants and 24 implants with artificially created fractures. Images were evaluated in their original and Canny formats. The accuracy of each radiograph was assessed by comparison with a reference standard of direct observation of the implant. The greatest area under the receiver operating characteristic curve belonged to Canny CBCT with MAR (0.958), followed by original CBCT with MAR (0.917), original CBCT without MAR = Canny CBCT without MAR = Canny OPA (0.875), Canny PPA (0.833), original PPA = original OPA (0.792), respectively. The Canny edge detection algorithm is suggested as an innovative method for accurate diagnosis of clinically suspected implant fractures on CBCT and periapical radiographies.

## Introduction

The use of dental implants for the treatment of partially or completely edentulous patients has gained popularity owing to its high success rate. As the use of dental implants increases, the associated complications also become more frequently encountered. Implant failure generally occurs due to secondary infection and/or undesirable biomechanical conditions. Fracture of the body of the implant is a relatively rare complication with an estimated incidence of 0.2–2.3%^1–5^. Despite its rarity, implant fracture is one of the most serious complications since it often necessitates removal of the fractured fixture which results in severe bone defects around the removal site^6–11^. In most cases, fractures secondary to bone loss and bone loss secondary to fractures are hard to distinguish; therefore, the diagnosis of implant fracture might be delayed if the clinician solely relies on the bone loss as a fracture sign. This highlights the role of radiographs as a non-invasive method of early diagnosis of implant fractures^12–15^.

Among conventional radiographic techniques, parallel periapical (PPA) radiographs are widely used for post-operative evaluation of dental implants. In this technique, the image receptor is positioned parallel with the implant and the incident beam is directed at right angles to the implant and the image receptor. PPA views have excellent spatial resolution; however, they could easily mask fractures that are not oriented in the direction of the incident beam. This problem can partly be overcome by altering the projection angle of the incident beam and taking oblique periapical (OPA) radiographs^16^.

As a 3D imaging technique, cone-beam computed tomography (CBCT) is the modality of choice for various purposes in dentistry including pre- and post-operative imaging of dental implants^17,18^. CBCT provides volumetric images with reliable details; however, beam hardening artifacts that are produced by high density objects such as titanium implants could degrade image quality to a point that diagnostic tasks including fracture diagnosis become almost impossible^19–21^.

Identifying an accurate method for the detection of dental implant fractures is of utmost importance since it could aid in the early diagnosis of the fractures, thereby reducing the amount of bone loss and the need for subsequent sophisticated treatments. Nevertheless, no studies so far have investigated the accuracy of different radiographic techniques for the diagnosis of implant body fractures. In the present study, we also tested an auto-edge detection-based technique to determine whether fracture diagnosis could be facilitated in the radiographic images of dental implants. The Canny edge detection algorithm is an accurate auto-edge detection method which defines the outline of an object and has the capability of detecting sharp intensity changes in an image^22,23^. Following the STARD guidelines^24^, we aimed to determine the diagnostic accuracy of PPA, OPA, and CBCT images with and without applying the Canny edge detection algorithm for the diagnosis of simulated implant fractures. The null hypothesis (H0) was that no significant difference exists in the diagnostic accuracy of original and Canny images for the detection of implant fractures.

## Methods

### Study design and preparation of the samples

This experimental study was performed *in-vitro* and approved by the Research Ethics Committee of Guilan University of Medical Sciences (Approval ID: IR.GUMS.REC.1401.010). All experiments were performed in accordance with relevant guidelines and regulations of the Declaration of Helsinki. Blocks of fresh bovine rib measuring 6 × 20 × 20 mm were prepared for simulation of the alveolar bone. The samples were gathered from the bone waste of a butcher shop and the animals were not directly involved at all. Initially, digital periapical radiographs were taken from the bone blocks to exclude the ones with cortical defects or abnormal trabecular patterns.

**Figure 1:**
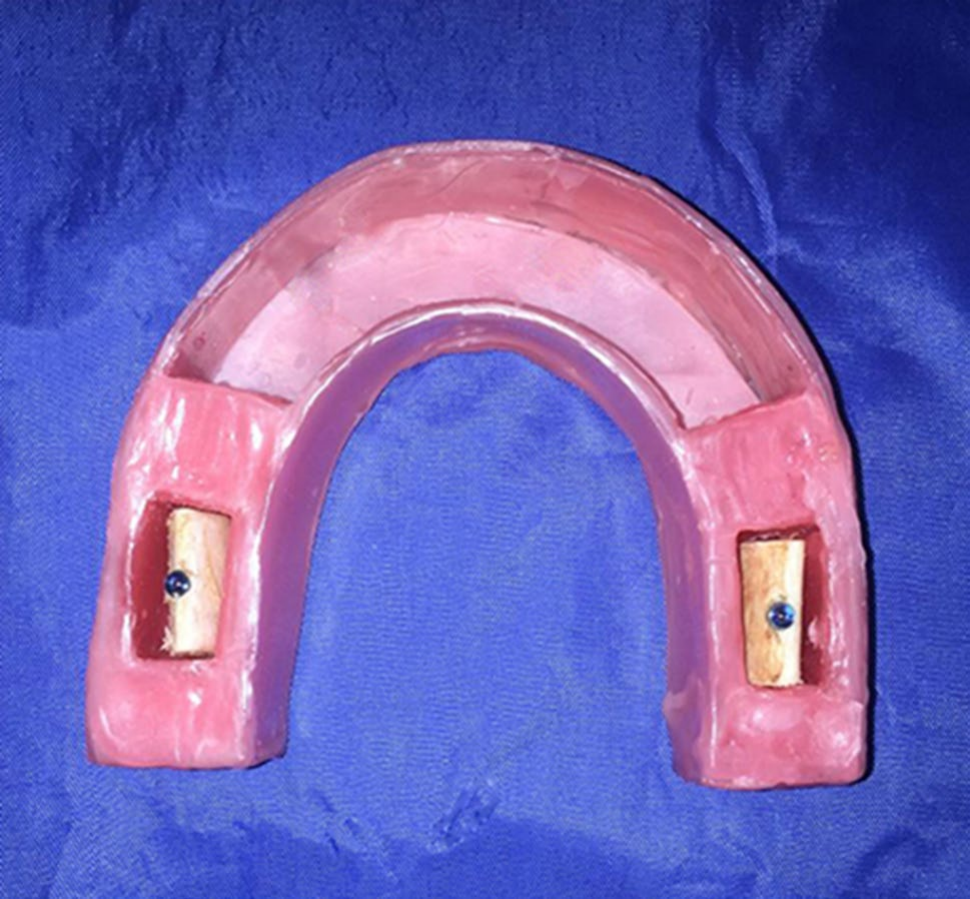
Wax model containing insertion sites for bone blocks with implants.

48 implants with the dimensions of 4 × 10 mm (SIC invent; Basel, Switzerland) were used. Simulated fractures were created in half of the implants using a diamond disk of ø 22 × 0.30 mm (Prodont- Holliger Speedy; Vence, France) by placing the disk at the third cervical thread of the implants and entering 1.5 mm deep inside the fixtures. In this way, fracture planes were created at the same position and with similar dimensions. The other 24 implants were remained intact to serve as the control group. The implants were inserted inside the bone blocks so that each block contained one implant. Cover screws were also placed on the fixtures. A wax arch was fabricated in the form of the mandibular arch with two spaces in its right and left posterior parts to serve as the object positions of bone block insertion (Fig. 1). The intact and fractured implants in the bone blocks were randomly placed within the wax arch so that only the radiologist who performed the radiographic examinations was aware of the fracture condition of the samples.

### Radiographic examinations

Three radiographic modalities including PPA, OPA, and CBCT were performed for all the samples. In order to obtain PPA views, a photostimulable phosphor plate (Digora Optime; Soredex, Tuusula, Finland) was placed completely parallel with the bone block and the central beam was directed at right angles to the bone block and the implant using an intraoral X-ray device (Minray; Soredex, Tuusula, Finland) at exposure settings of 70 kV, 7 mA, and 0.32 S. OPA radiographs were taken similar to the PPA views except for the central beam which was shifted 20° mesially relative to the implant. CBCT examinations were performed with the wax arch positioned at the center of the unit’s chin rest (Pax-i 3D; Vatech, Yongin, Korea). The exposure parameters included 95 kV, 5.2 mA, a FOV measuring 90 × 120 mm^2^, and a voxel size of 0.2 mm. For each pair of implants in the wax arch, the CBCT examinations were performed once in the active metal artifact reduction (MAR) mode and once in the inactive mode.

### Image processing

The PPA and OPA images were processed and saved by the Scanora imaging software version 4.3.1 (Digora Optime; Soredex, Tuusula, Finland). CBCT scans were automatically reconstructed (EZ3D-i; Vatech, Yongin, Korea). Cross-sectional views were created for each sample using the “section” tool of the software. Subsequently, the section that best displayed the whole length of the implant was selected and saved. In this way, 4 original-format images (PPA, OPA, CBCT with MAR on, and CBCT with MAR off) were obtained from each implant by the same radiologist who also acquired the radiographs. Afterwards, the images were processed in order to extract their edges. To do so, the Canny edge detection algorithm was used. In this algorithm, firstly, the image noise is removed by a Gaussian filter. Then, by applying a gradient magnitude thresholding technique, the edges of the image are detected. In the present study, based on the observations made by two expert radiologists, different values for the standard deviation of the Gaussian filter and the high and low thresholds were selected for processing the CBCT and periapical images. The aforementioned values were selected by two radiologists at a point where image details were displayed with the greatest fidelity. For the CBCT images the standard deviation of the Gaussian filter was set to 1.2, and 0.07 and 0.028 were used as the high and low thresholds, respectively. For the periapical images, the corresponding values of the parameters were set to 2.0, 0.18, and 0.072, respectively. All image processing stages were performed by codes developed in MATLAB 2018a (Math Works; Natick, MA, USA) computing platform.

**Figure 2:**
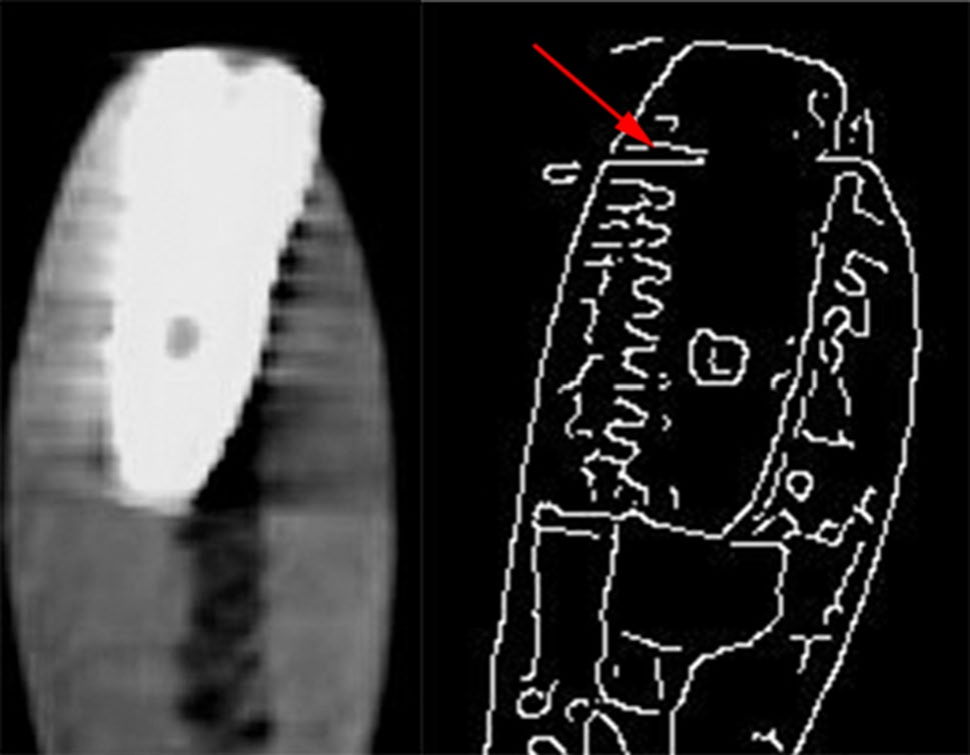
CBCT section of a fractured implant and its related Canny image (Arrow points to the fracture area which cannot be detected on the original image).

**Figure 3:**
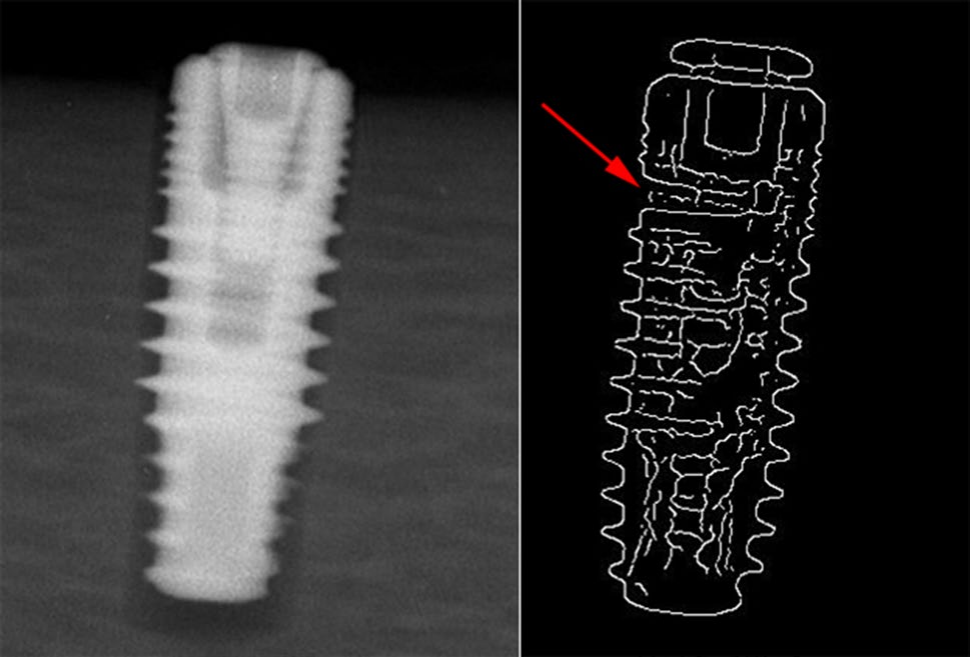
PPA view of a fractured implant and its related Canny image (Arrow points to the fracture area which cannot be detected on the original image).

Hence, 8 images (4 original and 4 with the applied Canny algorithm) were available as index tests for each sample. Since 24 intact and 24 fractured implants existed, a total number of 384 images were obtained from the study samples. The images were coded with random numbers to be viewed by three observers (two maxillofacial radiologists and a periodontist) who were totally unaware of the condition of the samples. Random numbers ensured that no bias occurred during the image obsevations. The observers were asked to record their diagnoses regarding whether each implant was intact or fractured (Figs. 2 and 3).

### Analysis

The diagnostic accuracy of the index tests (original and Canny images) against the reference standard (direct observation) is reported following the STARD guidelines^24^. MedCalc statistical software version 20.026 (MedCalc; Ostend, Belgium) was used to calculate index tests sensitivity and specificity, and also to perform and plot receiver operating characteristic (ROC) analysis as well as 95% confidence intervals. Confidence interval and standard error were defined by the exact binominal and DeLong tests, respectively. The optimal cut off value for sensitivity and specificity calculation was defined by the Youden method. Furthermore, pairwise comparisons were performed by the DeLong method with the significance level set at 0.05.

Inter-observer reliability was determined by the SPSS software version 28 (IBM; Armonk, NY, USA).

## Results

The inter-observer agreement was reliable between each pair of the observers since the kappa statistic was above 0.7. Sensitivity, specificity, and AUC of the 8 image types for fracture diagnosis are presented in Table 1. The significance values in Table 1 refer to the comparison of each method with the random technique. The random technique is defined as a method in which the chance of correct response is 50%. From Table 1, it is understood that all image types were significantly more accurate than the random technique. In other words, the chance of correct diagnosis in all modalities was higher than 50%.

**Table 1:** Diagnostic accuracy of the different image types for detection of simulated implant fractures.

Image type	AUC	SE^a^	95% CI^b^	*p* value	Sensitivity	Specificity
PPA original	0.792	0.051	0.65–0.89	< 0.001	0.58	1.00
OPA original	0.792	0.051	0.65–0.89	< 0.001	0.58	1.00
CBCT MAR (on) original	0.917	0.041	0.80–0.98	< 0.001	0.92	0.92
CBCT MAR (off) original	0.875	0.045	0.75–0.95	< 0.001	0.75	1.00
PPA Canny	0.833	0.049	0.70–0.92	< 0.001	0.67	1.00
OPA Canny	0.875	0.045	0.75–0.95	< 0.001	0.75	1.00
CBCT MAR (on) Canny	0.958	0.029	0.86–0.99	< 0.001	1.00	0.92
CBCT MAR (off) Canny	0.875	0.045	0.75–0.95	< 0.001	0.75	1.00

AUC area under the ROC curve, ^a^DeLong test, ^b^exact binominal test, *SE* standard error, *CI* confidence interval.

**Figure 4:**
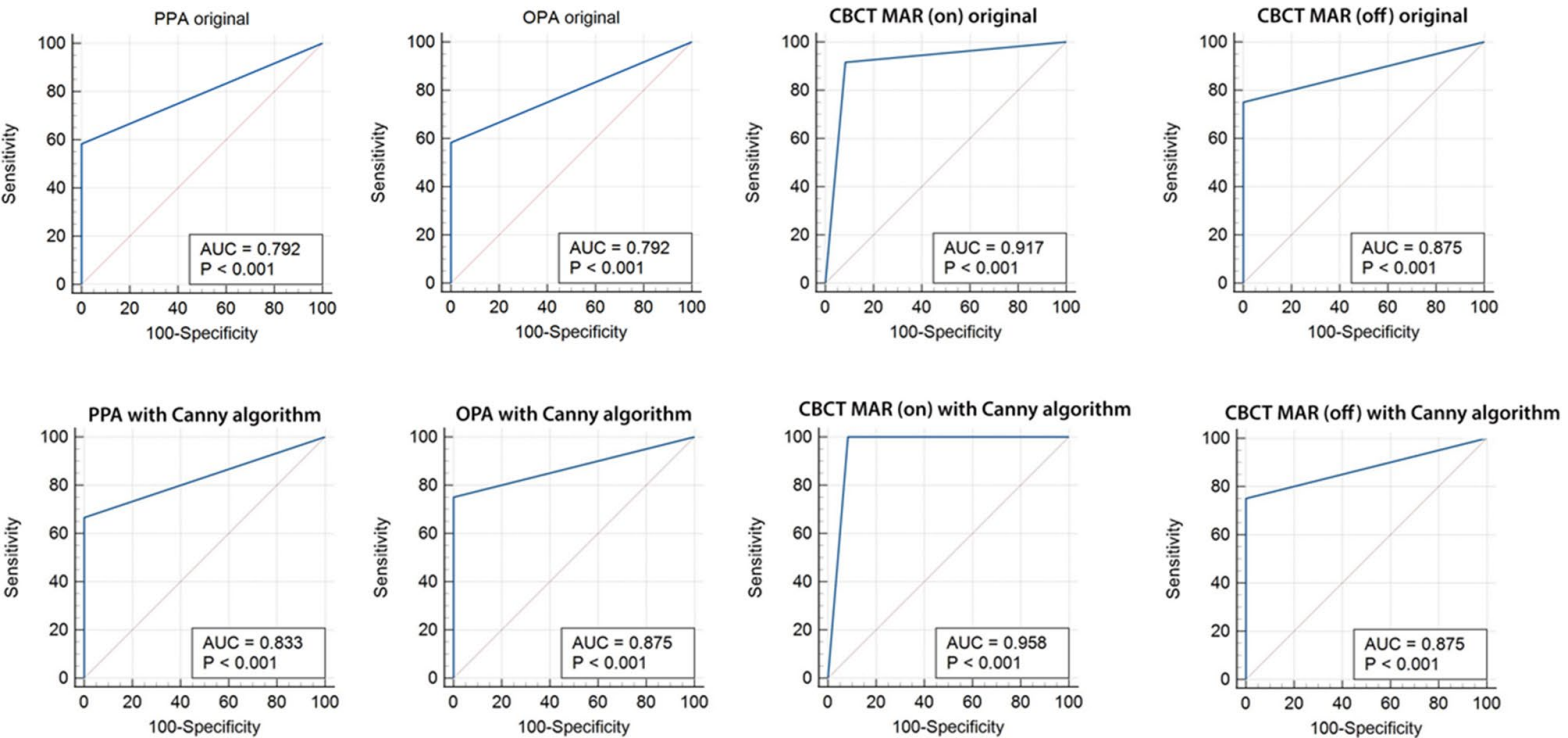
ROC curves related to the different image types and their comparisons with the random technique.

**Figure 5:**
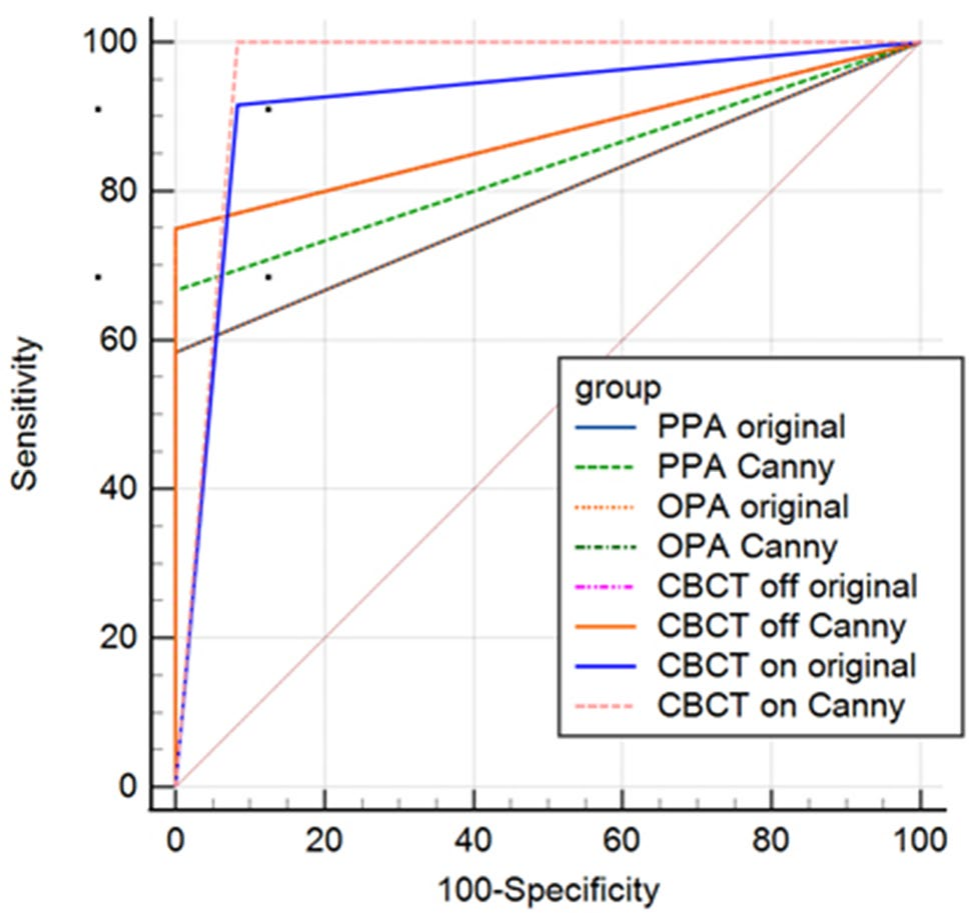
Comparison of the AUC among various image types.

Figure 4 shows the ROC curves of the different imaging modalities. By comparing the AUC values among the different image types which is also depicted on Fig. 5, it is clear that the highest value belongs to CBCT MAR (on) with Canny algorithm (0.958) and the lowest amount is related to the PPA original and OPA original images (0.792).

Pairwise comparisons were also made between each set of the radiographic images and showed that the differences in the AUC values were statistically significant between the following pairs of images: CBCT MAR (on) with Canny and PPA original (*p* = 0.005), CBCT MAR (on) with Canny and PPA with Canny (*p* = 0.028), and CBCT MAR (on) with Canny and OPA original (*p* = 0.005). The highest diagnostic accuracy was related to CBCT in the active MAR mode and applied Canny algorithm. Table 2 presents a thorough description of the pairwise comparisons.

**Table 2:** Paired comparisons of the diagnostic accuracy of the different radiographic techniques for fracture diagnosis in the implants.

	Difference in AUC	95% CI	*p* value ^a^
PPA original ~ PPA Canny	0.042	− 0.10 to 0.18	0.558
PPA original ~ OPA original	0	− 0.14 to 0.14	> 0.999
PPA original ~ OPA Canny	0.083	− 0.05 to 0.22	0.223
PPA original ~ CBCT MAR (off) original	0.083	− 0.05 to 0.22	0.223
PPA original ~ CBCT MAR (off) Canny	0.083	− 0.05 to 0.22	0.223
PPA original ~ CBCT MAR (on) original	0.125	− 0.004 to 0.25	0.057
PPA original ~ CBCT MAR (on) Canny	0.167	0.05 to 0.28	0.005*
PPA Canny ~ OPA original	0.042	− 0.10 to 0.18	0.558
PPA Canny ~ OPA Canny	0.042	− 0.09 to 0.17	0.532
PPA Canny ~ CBCT MAR (off) original	0.042	− 0.09 to 0.17	0.532
PPA Canny ~ CBCT MAR (off) Canny	0.042	− 0.09 to 0.17	0.532
PPA Canny ~ CBCT MAR (on) original	0.083	− 0.04 to 0.21	0.192
PPA Canny ~ CBCT MAR (on) Canny	0.125	0.01 to 0.24	0.028*
OPA original ~ OPA Canny	0.083	− 0.05 to 0.22	0.223
OPA original ~ CBCT MAR (off) original	0.083	− 0.05 to 0.22	0.223
OPA original ~ CBCT MAR (off) Canny	0.083	− 0.05 to 0.22	0.223
OPA original ~ CBCT MAR (on) original	0.125	− 0.004 to 0.25	0.057
OPA original ~ CBCT MAR (on) Canny	0.167	0.05 to 0.28	0.005*
OPA Canny ~ CBCT MAR (off) original	0	− 0.12 to 0.12	> 0.999
OPA Canny ~ CBCT MAR (off) Canny	0	− 0.12 to 0.12	> 0.999
OPA Canny ~ CBCT MAR (on) original	0.042	− 0.08 to 0.16	0.493
OPA Canny ~ CBCT MAR (on) Canny	0.083	− 0.02 to 0.19	0.120
CBCT MAR (off) original ~ CBCT MAR (off) Canny	0	− 0.12 to 0.12	> 0.999
CBCT MAR (off) original ~ CBCT MAR (on) original	0.042	− 0.08 to 0.16	0.493
CBCT MAR (off) original ~ CBCT MAR (on) Canny	0.083	− 0.02 to 0.19	0.120
CBCT MAR (off) Canny ~ CBCT MAR (on) original	0.042	− 0.08 to 0.16	0.493
CBCT MAR (off) Canny ~ CBCT MAR (on) Canny	0.083	− 0.02 to 0.19	0.120
CBCT MAR (on) original ~ CBCT MAR (on) Canny	0.042	− 0.06 to 0.14	0.404

*AUC* area under the ROC curve, *CI* confidence interval, ^a^DeLong test.

Significant values are in bold.

## Discussion

The null hypothesis (H0) was rejected since significant differences were observed between the original and Canny images in terms of fracture diagnosis.

Fracture of the body of dental implants rarely occurs; however, it is one of the most serious complications of implant therapy. Several factors have been proposed as the risk factors of implant fracture, among which implant design and diameter, implant length, implant-abutment misfit, treated area, long lasting time, parafunctional activities, occlusal overload, and type of prosthesis are the most important^2,25–28^. In many cases, it cannot be determined whether marginal bone resorption is a cause of implant fracture or a consequence of it^12^. This means that implant fracture can occur prior to bone loss in some cases and hence it is necessary to diagnose the fracture in a timely manner in order to minimize the amount of bone defect that occurs around the fractured implant and therefore the need for future augmentation procedures. Several studies have been performed on the prevalence and related risk factors of implant fracture^1,11,29,30^; nevertheless, no attempts so far have been made to identify the best radiographic modality for accurate diagnosis of fractures in dental implants. Since the radiodensity and geometric configuration of dental implants are very different from natural teeth, a survey on different radiographic methods is necessary to define the best technique.

To the best of our knowledge, this is the first study to compare the diagnostic accuracy of various radiographic methods for the detection of simulated fractures in dental implants. We evaluated CBCT and periapical radiographies with the former in two active and inactive modes of MAR and the latter in two forms of parallel and oblique angle of X-ray projection. Simulated fractures were created in the cervical portion of the fixtures since it is the most load-bearing area and therefore more susceptible to fractures^31^. However, it should be noted that true fractures differ from the artificial fractures of our study in terms of size and location. True fractures are usually narrower and occur at the implant shoulder extending down vertically^2^.

Another distinguishing feature of this study was the application of an auto-edge detection algorithm (Canny algorithm) on the radiographic images to determine whether it facilitates diagnosis of the fractured samples.

Periapical radiographs are routinely used for the post-operative assessment of dental implants. Furthermore, they are frequently used for diagnosis of root fractures due to their excellent spatial resolution. However, the two-dimensional nature of these radiographs makes it almost impossible to diagnose the fractures that are not oriented in the direction of the central X-ray beam. Thus, it is highly recommended that a second radiograph with different angulation be taken when a fracture is doubted^16^. In the present study, we assessed both PPA and OPA radiographies for diagnosis of the simulated fractures. PPA views were obtained with the central beam directed at right angles to the implant and the image receptor while OPA images were obtained by shifting the central beam 20° in the horizontal plane. The diagnostic accuracy of the two techniques were exactly the same when they were obtained in their original formats (AUC = 0.792). In the present study, the simulated fractures involved three planes of the fixtures (one proximal and two facio-lingual surfaces); hence, the similar accuracy of PPA and OPA radiographs could be related to the involvement of the proximal surfaces which can be properly displayed in both modalities. Saberi et al.^32^ reported that the use of OPA images greatly enhances the visibility of fenestration and dehiscence defects when they are confined to the buccal aspect of dental implants. This finding further highlights the importance of the surfaces that are involved in fractures or bony defects around the implants.

CBCT images were taken in two MAR (on) and MAR (off) modes, both modes having greater accuracy compared to the PPA and OPA images. Previous studies have reported that CBCT images are superior to periapical radiographs for the detection of root fractures^16,33^. In the present study, we found that the same fact also exists for simulated implant fractures. Although interfering artifacts are produced in the vicinity of titanium implants, it seems that the three-dimensionality of the CBCT images outweighs the undesirable effects of these artifacts. However, activation of the MAR mode obviously resulted in more accurate diagnosis of the simulated implant fractures (AUC = 0.917 for MAR on mode *vs.* AUC = 0.875 for MAR off mode). Kajan et al. and Candemil et al., also observed that application of the MAR tool in the CBCT images enhances the detectability of vertical root fractures particularly when the teeth contain intracanal metallic posts^34–36^.

Computer-aided systems are gaining popularity in medical and dental imaging diagnosis. These techniques could be applied to nearly all types of radiographic images to enhance the accuracy and feasibility of different diagnostic tasks^37^. A unique feature of the present study was the application of an auto-edge detection algorithm, i.e. the Canny algorithm for identification of the simulated implant fractures. Canny edge detection algorithm is an accurate tool for defining the outline features of an object as well as detecting sharp intensity changes in an image. The algorithm works in MATLAB computing platform and has been used in medical imaging for expressing bone changes of osteoporosis and also artifact quantification in CT images^22,23^. Canny edge detection algorithm benefits from being less sensitive to sources of image noise including gray level inhomogeneity caused by exposure conditions, patient’s position, and environmental temperature^38^. Since it has the capability of defining the intensity changes and sharp edges of an image with the least amount of noise, we decided to apply it on the radiographic images to determine whether it enhances the accuracy of the radiographic images for implant fracture diagnosis. Three parameters of the Canny algorithm including standard deviation of the Gaussian filter, high, and low sensitivity thresholds can be adjusted manually by the operator. In this study, two expert radiologists who were not among the observers defined the aforementioned parameters in the CBCT and periapical images by testing different values until they both reached a point with the least amount of noise and finest image details.

By applying the Canny edge detection algorithm on the images, we obtained higher amounts of diagnostic accuracy in the PPA, OPA, and CBCT MAR (on) images. This means that regardless of the two- or three-dimensional nature of the radiographic images, application of the Canny algorithm enhances the visibility of simulated implant fractures owing to the sharp intensity changes it displays. By looking at the specificity values in Table 1, we come to another important feature of the Canny algorithm which is application of this algorithm on the radiographic images does not give rise to the number of false positive results.

The AUC values were similar for the original and Canny formats of the CBCT MAR (off) images. This finding could be suggestive of lower efficiency of the Canny algorithm in the presence of metal artifacts. Therefore, it can be concluded that the Canny algorithm creates more accurate images when the metal artifacts are reduced or eliminated.

By comparing the AUC values, it is perceived that the highest accuracy belongs to Canny CBCT with MAR, followed by original CBCT with MAR, original CBCT without MAR = Canny CBCT without MAR = Canny OPA, Canny PPA, original PPA = original OPA, respectively. Pairwise comparisons also revealed statistically significant differences between Canny CBCT with MAR and original PPA (*p* = 0.005), Canny CBCT with MAR and Canny PPA (*p* = 0.028), and Canny CBCT with MAR and original OPA (*p* = 0.005).

There are also limitations to the present study; most important of all being the configuration of the fractures. Since the present study was performed *in-vitro*, fractures were created with standardized outlines. However, in actual situations fractures occur with more bizarre shapes that may influence the diagnostic accuracy. In the present study, simulated fractures were 0.3 mm wide and they were created horizontally at the cervical portions of the fixtures, while true fractures are usually narrower which may limit the voxel resolution of CBCT images to display them properly. Moreover, true fractures mostly occur at the implant shoulder extending down vertically^2^. Thus, it is recommended that further clinical studies be carried out to define the efficiency of various radiographic modalities as well as the Canny edge detection algorithm for diagnosis of true implant fractures.

## Conclusion

Application of the Canny edge detection algorithm on periapical and CBCT images significantly enhances the accuracy of simulated implant fracture diagnosis. CBCT displays implant fractures more precisely compared to periapical radiography particularly when the MAR tool is activated and the Canny algorithm is also applied. Clinical studies with real conditions in terms of fracture size and orientation are recommended to test the diagnostic accuracy of the imaging techniques and algorithmic functions.

## Data Availability

The data that support the findings of this study are available on request from the corresponding author.
